# Helical Nanostructures of Ferroelectric Liquid Crystals as Fast Phase Retarders for Spectral Information Extraction Devices: A Comparison with the Nematic Liquid Crystal Phase Retarders

**DOI:** 10.3390/ma14195540

**Published:** 2021-09-24

**Authors:** Marwan J. AbuLeil, Doron Pasha, Isaac August, Evgeny P. Pozhidaev, Vadim A. Barbashov, Timofey P. Tkachenko, Artemy V. Kuznetsov, Ibrahim Abdulhalim

**Affiliations:** 1 Department of Electrooptic and Photonics Engineering, Ilse Katz Institute of Nanoscale Science and Technology, ECE-School, Ben Gurion University of the Negev, Beer Sheva 84105, Israel; abuleil@post.bgu.ac.il (M.J.A.); doronpas@post.bgu.ac.il (D.P.); 2 Department of Electrical Engineering and Physics, Sami Shamoon College of Engineering, Beer Sheva 8410802, Israel; isaacau@ac.sce.ac.il; 3 P. N. Lebedev Physical Institute of the Russian Academy of Sciences, Leninsky Pr. 53, 119991 Moscow, Russia; pogidaevep@lebedev.ru (E.P.P.); vadbar13@lebedev.ru (V.A.B.); tptkachenko@lebedev.ru (T.P.T.); kuznetsovav@lebedev.ru (A.V.K.)

**Keywords:** liquid crystal devices, compressed sensing, inverse scattering, spectral imaging

## Abstract

Extraction of spectral information using liquid crystal (LC) retarders has recently become a topic of great interest because of its importance for creating hyper- and multispectral images in a compact and inexpensive way. However, this method of hyperspectral imaging requires thick LC-layer retarders (50 µm–100 µm and above) to obtain spectral modulation signals for reliable signal reconstruction. This makes the device extremely slow in the case of nematic LCs (NLCs), since the response time of NLCs increases proportionally to the square of the LC-layer thickness, which excludes fast dynamic processes monitoring. In this paper, we explore two approaches for solving the speed problem: the first is based on the use of faster nanospiral ferroelectric liquid crystals as an alternative to NLCs, and the second is based on using a passive multiband filter and focuses on multispectral extraction rather than hyperspectral. A detailed comparative study of nematic and ferroelectric devices is presented. The study is carried out using a 9-spectral bands passive spectral filter, covering the visible and near-infrared ranges. We propose the concept of multispectral rather than hyperspectral extraction, where a small number of wavelengths are sufficient for specific applications.

## 1. Introduction and Motivation

Liquid crystals (LCs) can be considered as one of the most important functional materials of the current century, since liquid crystal displays that demonstrate high contrast, high resolution full-color images, compactness, and relatively low cost are currently the mainstream of information displays [1]. In the last three decades, there is an ongoing tendency of decreasing the response time of LCs with a simultaneous decrease in their energy consumption for use in other photonic rather than display applications [2,3]. The most mentioned photonic structure over the past few years for use in biomedicine is optical tweezers [4], for which the Nobel Prize in Physics for 2018 was awarded [5]. A decade and a half after the discovery of optical tweezers, the concepts of holographic optical tweezers based on liquid crystal phase matrices were proposed and experimentally confirmed [6,7,8]. Holographic optical tweezers based on a nematic phase matrix (spatial light modulator-SLM) were described in [6,7], and a similar device was considered in [8], but based on a surface-stabilized ferroelectric liquid crystal (FLC) [9,10], which switches 30–40 times faster than a similar device based on nematic LC (NLC). In addition to optical tweezers, biomedical LC applications include LC biosensors and portable wearable devices on the patient’s body for diagnostic and medical imaging [11]. Among them are customizable filters for multispectral and hyperspectral images, polarization monitoring devices for polarimetric images, and SLMs that provide a variety of visualization modes of health state parameters [12,13,14]. The potential of LC thermography of the skin was investigated to detect skin and breast cancer [15,16], as well as to diagnose thrombosis [17,18].

**Figure 1:**
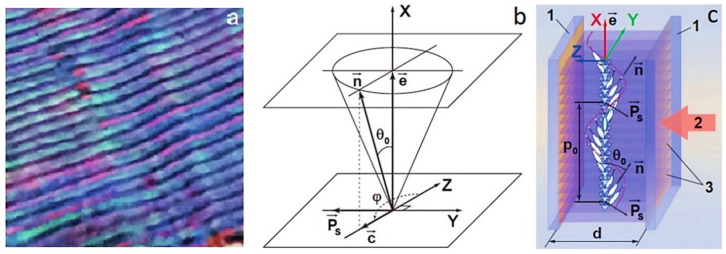
(**a**) An image of the HSFLC in a polarizing microscope, image size is 80 μm × 80 μm [19]; (**b**) the location of the HSFLC director $\vec{n}$ in a separate smectic layer, the plane of which is parallel to YZ plane, ${\vec{P}}_{s}$ is the spontaneous polarization vector, $\vec{c}$ is c-director, $\vec{e}$ is the normal to the smectic layer plane, *θ*_0_ is the molecular tilt angle in smectic layers, *φ* is azimuth angle of the director in the plane of the smectic layer [20]; (**c**) the structure of HSFLC bounded by flat transparent and conductive substrates 1. Arrow 2 shows the direction along the planes of the smectic layers 3, which coincides with the direction of light propagation. The thickness of the HSFLC is designated as *d* [21].

All the above-mentioned concepts of biomedical applications of LCs have been developed for NLCs, and the potential of photonic applications of helical nanostructures of ferroelectric liquid crystals (HNFLCs) has not yet been revealed. However, this type of self-organizing LC nanostructures was studied quite well. The helical structure of a ferroelectric liquid crystal (HSFLC) was first discovered in [9], where it was reported about the discovery of ferroelectricity in liquid crystals but the helix pitch *p*_0_ varied from 5 μm to 10 μm. Such a structure can be visually observed in a polarizing microscope [19] (see Figure 1a) if the helix axis is parallel, and the planes of the smectic layers are perpendicular to the planes of the substrates (Figure 1b,c explain the structure geometry [20,21]).

The pitch *p*_0_ of the helix strongly depends on the molecular structure. The most effective control of *p*_0_ value is possibly due to the chiral esters of terphenyl dicarboxylic acid [22,23]. Depending on the homologue number and the concentration of these structures in liquid-crystal mixtures, the pitch of the HNF and antiferroelectric LCs (AFLC) helix can vary from 50 nm [24] to infinity [25].

Apparently, it is legitimate to speak about the helical nanostructures of ferroelectric LCs, if the pitch of the helix is less than 500 nanometers. The helix pitch of the order of 300 nm to 500 nm is typical for smectic C* mixtures of chiral lactic acid esters [26]. Helical configurations of ferroelectric and antiferroelectric LCs exhibit a very wide range of optical and electro-optical phenomena. Even in the simplest case of light propagation along the helix axis, many optical effects are observed [27], in particular, circularly polarized Bragg reflection, a huge optical rotational dispersion, optical solitons, and the adiabatic following phenomenon in the Mauguin limit [28]. The possibility of using these effects to create tunable filters, in thermography, as one-dimensional photonic crystals for LC lasers when operating near the edge of the photonic band gap [29], in electrically tunable optical diodes [30] has been proved. When deformed under an external field the helical nanostructure of an antiferroelectric LC, optical phase grating arises [31], which can be controlled by the field in which the diffraction orders are rearranged with response time of about 200 microseconds.

The electro-optical effect of the helical nanostructure of a FLC [32] and AFLC [24] deformation in an electric field (DHF-effect) is known for a long time. When light propagates along the axis of the helix, this effect provides a pure phase modulation of light under applied voltage, similar to the B effect in nematic LCs, however two orders of magnitude faster [33,34]. Note that the time of the electro-optical response in the DHF effect is proportional to the product of the rotational viscosity and the square of the helix pitch [32], therefore minimizing these parameters is one of the key materials science problems of helical nanostructures of FLCs and AFLCs. When light propagates perpendicular to the helix axis, the deformation of the helical nanostructure of a FLC in an external electric field ensures a smooth restructuring (in the entire visible spectral range) of the radiation spectrum passing through a single cell located between crossed polarizers [35,36]. To create a continuously tunable Lyot filter, just two DHF cells are enough [37], while the passband offset of the filter under the action of a voltage pulse is 185 microseconds.

Thus, the analysis of the literature data shows that there are all the necessary prerequisites for starting the development of the physico-technological and physico-chemical bases for the creation of optical devices for medical diagnostics based on helical nanostructures of FLCs and AFLCs. However, Lyot-based filters are bulky because a cascade of devices is needed and therefore cannot be wearable to monitor health parameters in real time. Single devices such as Fabry–Perot tunable filter [38] or the guided mode resonance [39] can be compact and wearable, however their manufacturability is difficult and their dynamic range is limited to 50 nm to 100 nm at most. A combination of small number of Lyot stages with a passive filter of transmission function composed of multiple bands allowed obtaining discrete tunability over large number of narrow bands in a faster manner and higher throughput [40], however, it still inadequate for wearable solutions. A single LC layer between two substrates was used recently as a spectral modulator with its known transfer function between two polarizers at many voltages [41]. The transfer function versus voltage is used as a known matrix to solve the inverse problem of finding the spectral information on an object within a scene.

To solve the problem efficiently compressed sensing algorithms were used. However, with this approach the LC layer must be thick in the order of 50 µm to100 µm at least in order to get transfer function rich in peaks and valleys. When NLCs were used for this purpose, the speed becomes slow of the order of minutes or more depending on the number of voltages required, the larger the better spectral resolution. The main purpose of the present paper is to highlight the potential of the fast nano-helical FLCs (nh-FLCs) instead of NLCs as the spectral modulation devices and use the compressed sensing or other inverse scattering approaches to resolve the inverse problem. As a first step we investigate a single nh-FLCs retarder between two polarizers combined with a passive filter having a transmission function composed of 9 narrow bands. Starting from the multispectral case simplifies the computation and relaxes the requirement on the retarder thickness. In many applications the hyperspectral information is not required for example in skin cancer diagnosis and pulse oximetry. Being compact and fast this device can be worn on the skin in the form of wristwatch or other forms to provide large spectral information of light scattered from the tissue within the time frame of a heartbeat, that is typically 1 s. Other possible application is in cancer diagnosis where sometimes it is enough to use a small number of wavelengths that characterize certain number of chromophores [42]. This approach can also be used for blood analytes sensing by measuring the scattered spectrum from the tissue or the auto-fluorescence from the skin by following the shift in some characteristic narrow spectral signatures, see Table 1.

If one needs to find the minimum of the spectrum due to absorption such as a wide absorption band, we can use an etalon or a passive filter that provides narrow spectral lines closely separated covering the whole region of the absorption band. As mentioned before, measuring PPG signals in real time and at many wavelengths has a great value for continuous monitoring of oxygen saturation levels, heartbeat, blood pressure, and possibly other parameters simultaneously. Pulse oximeters usually operate with two wavelengths, however in certain cases this was found to be not accurate enough for example with newborn infants. The use of PPG information from larger number of wavelengths should improve the accuracy. If fluorescence is measured such as auto-fluorescence from the skin then information on diabetes type 2, cardiovascular disease, and mortality can be predicted as it was shown recently [59]. Once a certain application is developed, the most important wavelengths can then be selected and used to measure, monitor, or diagnose either in medical, environmental, or industrial inspection applications. Usually, LEDs having different wavelengths are used serially, but the spectrum of LEDs is relatively wide with the narrowest being around 20 nm, however the use of narrow bands gives larger information. In Table 1 we list a selection of applications that require only small number of wavelengths.

## 2. Comparison of the Electrooptic Response of nh-FLCs with NLCs

**Figure 2:**
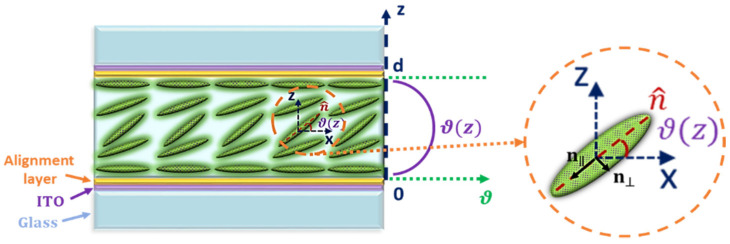
Schematic structure of anti-parallel aligned NLC device. The left side presents the NLC device structure, the right part shows a zoom for the molecular orientation according to the axes (assuming that the alignment direction is along the x axis) and $\vartheta \left(z\right)$ represents the director profile.

The main requirements from LCDs in the compressed sensing or other inverse scattering approaches can be summarized by short response time to extract spectrum in a reasonable time (within the time frame of a heartbeat), large phase shift to provide a wide variety of spectrum modulations, high contrast to overcome the noise, and low energy consumption. This section elucidates in short, the difference between DHFLCs and NLC devices structure and electrooptic response. The primary anti-parallel alignment LC device is described in Figure 2. In NLC with positive dielectric anisotropy, the molecules tend to orient with their long axis along the applied electric field direction without sign dependence as the interaction with the field is through the induced dipoles. Subsequently, the tilt angle ϑ of the director $\hat{n}$ changes along z axis (by assuming that z axis is normal to the substrate) and function of the voltage **V**. Then, the local LC birefringence changes due to the change in the effective extraordinary refractive index $n_{e}$ :

(1) $$\Delta n=n_{e}\left(\vartheta \left(z,V\right)\right)-n_{⊥}=\frac{n_{⊥}n_{∥}}{\sqrt{n_{∥}{}^{2}+\left(n_{⊥}{}^{2}-n_{∥}{}^{2}\right)cos^{2}\left(\vartheta \left(z,V\right)\right)}}-n_{⊥}$$

**Figure 3:**
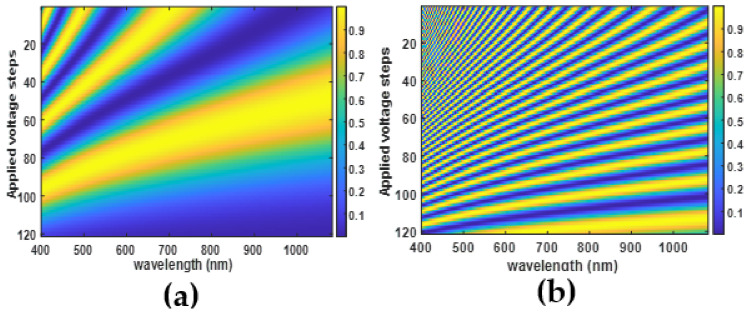
Simulation of spectral modulations of NLC devices using E7 NLC parameters at different applied voltages for two NLC thicknesses: (**a**) 5 μm and (**b**) 50 μm while the color of the fringes represents the transmission coefficient value.

Here $n_{⊥}$ , $n_{∥}$ are the refractive indices perpendicular and parallel to the director, respectively. For uniform director tilt angle, the total retardation can be determined using:

(2) $$\Gamma =\frac{2\pi \Delta nd}{\lambda }\;,$$

where d is the NLC layer thickness, and **λ** the light wavelength. De facto, the director’s tilt angle is nonuniform along the z axis, as Figure 3 describes. Then, total retardation can be calculated by:

(3) $$\Gamma =\frac{2\pi }{\lambda }\int_{0}^{d}(n_{e}\left(\vartheta \left(z,V\right)\right)-n_{⊥})\;dz\;\;\;$$

Approximate analytic expressions were proposed to simplify the design [60].

Commonly, the LCDs are fixed between two crossed or parallel polarizers to modulate the light spectrum while the LC optical axis is at 45 degrees to the first polarizer. Based on Jones calculations, the light transmission in the case of crossed polarizers is $T=sin^{2}\left(\Gamma ,/2\right)$ and $T=cos^{2}\left(\Gamma /2\right)$ for the case of parallel polarizers. Then, applying various electrical fields leads to different spectral modulations as Figure 3 shows. It is seen that increasing the LCD thickness to tens of micrometers increases the phase retardation and provides more variations in the modulations. For instance, Figure 3b shows that spectral modulation based on 50 µm NLC varies from large number of fringes (more than 20) at low applied voltages to one fringe at higher voltages. Hence it is an essential system requirement for spectral modulation and computational approach.

The response time of NLC is in the range of milliseconds to tens of milliseconds. The drawback here is that thick NLC retarders suffer from long response time, leading to a significant increase of the data acquisition time, exceeding 100 s in some cases [60,61]. Increasing the LC thickness d by a factor of 2 increases the LCD response time by a factor of 4 [62] according to the equations below:

(4) $$\tau_{decay}=\frac{\gamma_{1}}{K}{\left(\frac{d}{\pi }\right)}^{2}\;$$

(5) $$\tau_{rise}=\frac{\tau_{decay}}{{\left(\frac{V}{V_{c}}\right)}^{2}-1}$$

where $\tau_{decay}$ , $\;\tau_{rise}$ represent the molecules dynamics decay and rise times, respectively. $V_{c}$ *,* $\gamma_{1},$ and K represent threshold voltage, rotational viscosity, and reduced elastic constant (**K** *=* **K**_11_ *=* **K**_22_ *=* **K**_33_), respectively. Equations (4) and (5) assume infinite anchoring and the device can become slower when under finite anchoring conditions. To overcome this obstacle, several methods were proposed to decrease the NLC response time, such as the overdrive/undershoot voltage technique and replacing thick LC retarder with two or more thinner LC retarders, which can also improve the field of view. The use of materials with low viscosity as well as the dual frequency mode are all techniques being used to improve the speed of NLCs. These techniques can reduce the response time to a few milliseconds [62]. Decreasing the LCDs response is necessary for applications that require fast data acquisition and processing, such as extracting the spectrum of reflected light from moving objects or during rapidly changing bio-signals. LCDs with response time in the range of microseconds are proper candidates for these applications.

**Figure 4:**
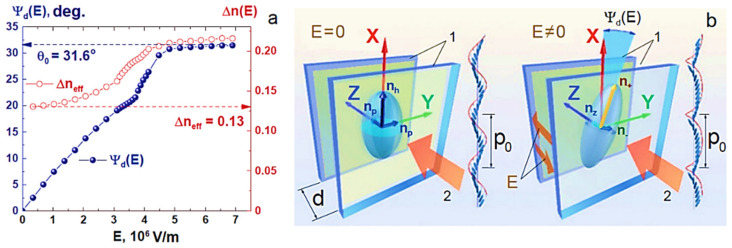
(**a**) Dependences on the electric field of the effective birefringence $\Delta n_{eff}\left(E\right)$ (**red circles**) and the deviation of the main optical axis $\Psi_{d}\left(E\right)$ (**blue balls**) of the planar-aligned liquid DHFLC-587-F7 with the helix pitch around 65 nm, developed in [73]. The measurements were carried out at 22 °C at a wavelength **λ** = 632.8 nm; (**b**) illustration of the biaxial transformation of the effective refractive index ellipsoid of the DHFLC in an electric field in a planar-aligned DHFLC cell (the same geometry as in Figure 1c). Numeral 1 denotes transparent conductive substrates, arrow 2 shows the direction of propagation of linearly polarized incident light, the same as in Figure 1c. All images are taken from [69].

FLCs are characterized by response time in the range of microseconds due to the presence of the spontaneous polarization ${\vec{P}}_{s}$ (Figure 1) that canceled out in NLC due to symmetry [63,64,65,66]. Sensitivity to the electrical field sign also stems from the spontaneous polarization existence. The distorted helix FLCs (DHFLCs) [32] can provide continuous modulation of the light intensity due to the continuous variations of the effective birefringence $\Delta n_{eff}\left(E\right)\sim E^{2}$ and the optical axis angle $\Psi_{d}\left(E\right)\sim E$ in electric field **E**, as it is illustrated in Figure 4a. The illustration denotes that the light intensity modulation with DHFLCs is described in terms of the classical optics of crystals, namely in terms of ellipsoids of the effective refractive indices (Figure 4b) that was grounded both theoretically and experimentally [33,34,67,68,69,70,71]. Note that this approach is valid when the effects of rotation of the plane of polarization of light and Bragg reflection in the helicoidal structure are not significant, which is the case if:

(6) $$p_{0}<\frac{\lambda }{5}$$

as it was predicted theoretically [72] and confirmed experimentally [69,71].

Biaxial medium behavior appears by homogenizing the dielectric tensor method for a small electrical field and birefringence expression $\Delta n_{eff}\left(E\right)$ is extracted [33,34,67,68,70,74,75] under the condition (6). The homogenization theory [67,68,70] shows that the main optic axis averages to be along the helix axis **X** at **E** = 0, see the left side of the Figure 4b. As the field is applied, the average optic axis rotates by angle $\Psi_{d}\left(E\right)$ in the substrates’ plane XY, and together with it the effective birefringence changes, see the right side of the Figure 4b. In this case the light transmittance **T_z_** through the DHFLC cell placed between crossed polarizers is determined by [67,68]:

(7) $$T_{z}=sin^{2}2\left[\beta +\Psi_{d}\left(E\right)\right]sin^{2}\frac{\Delta \Phi \left(E\right)}{2}$$

Here β is the angle between the first polarizer and x axis, and $\Delta \Phi \left(E\right)$ is the phase retardation, which is written as:

(8) $$\Delta \Phi \left(E\right)=\frac{2\pi d\Delta n_{eff}\left(E\right)}{\lambda }$$

and $\Delta n_{eff}\left(E\right)$ at **E** << **E_c_** (**E_c_** is the critical field of the helix unwinding [74]) is expressed as [33,34,67,75,76]:

(9) $$\Delta n_{eff}\left(E\right)=\Delta n_{u}\left(\lambda \right)\left[1-\frac{3}{2}sin^{2}\theta_{0}+sin^{4}\theta_{0}\frac{sin^{2}2\theta_{0}}{1-\frac{3}{2}sin^{2}\theta_{0}}{\left(\frac{\varepsilon_{0}\chi_{G}}{P_{s}}\right)}^{2}E^{2}\right]=\Delta n_{eff}^{0}+\Delta n_{eff}^{E}$$

where

(10) $$\Delta n_{eff}^{0}=\Delta n_{u}\left(\lambda \right)\left(1-\frac{3}{2}sin^{2}\theta_{0}\right)$$

(11) $$\Delta n_{eff}^{E}=\Delta n_{u}\left(\lambda \right)\left[sin^{4}\theta_{0}\frac{sin^{2}2\theta_{0}}{1-\frac{3}{2}sin^{2}\theta_{0}}{\left(\frac{\varepsilon_{0}\chi_{G}}{P_{s}}\right)}^{2}E^{2}\right]=K_{Kerr}\lambda E^{2}$$

Here, in (8)–(11) $K_{Kerr}$ is the orientational Kerr-effect coefficient, $\chi_{G}$ is dielectric susceptibility of the helical structure (Goldstone mode), $\varepsilon_{0}$ is dielectric permittivity of vacuum, $\Delta n_{u}\left(\lambda \right)=n_{e}\left(\lambda \right)-n_{o}\left(\lambda \right)$ is the FLC birefringence dispersion under condition of the complete helix unwinding, at **E > E_c_**; $n_{e}\left(\lambda \right)$ and $n_{o}\left(\lambda \right)$ are refractive indices dispersions along and perpendicular to long molecular axes, respectively. Typical dependencies on the electric field of the birefringence $\Delta n_{eff}\left(E\right)$ and the rotation angle $\Psi_{d}\left(E\right)$ of the optic axis can be seen in Figure 4a.

**Figure 5:**
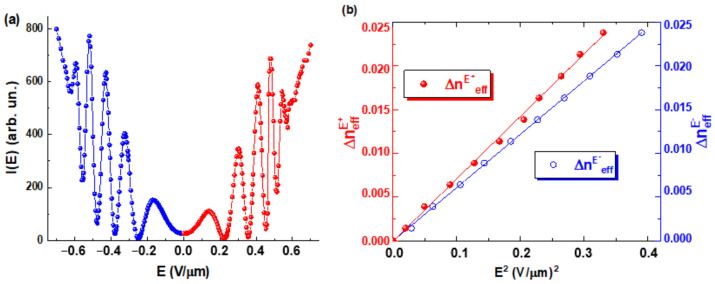
(**a**) measured intensity *I*(*E*) of monochromatic light (**λ** = 532 nm) transmitted through the DHFLC cell (*d* = 107 μm) placed between crossed polarizers at **β** = 0. Ferroelectric liquid crystal FLC-618 described in [34] was used in experiments carried out and at 23 °C, the applied electric field frequency 7 Hz; (**b**) the effective birefringence $\Delta n_{eff}^{E^{+}}$ and $\Delta n_{eff}^{E^{-}}$ of the FLC-618 versus square of the applied electric field for its both polarities: $\Delta n_{eff}^{E^{+}}$ (**red balls**) was measured for the positive polarity, while $\Delta n_{eff}^{E^{-}}$ (**blue circles**) for the negative one.

It is seen from the relation (7) and Figure 4a that the DHFLC retarder exhibits the simultaneous variations of its birefringence and the optical axis rotation versus the applied electric field. Because of this feature, the amplitude modulation coefficient of monochromatic radiation intensity I, transmitted through the DHFLC retarder placed between crossed polarizers, changes with the applied field (Figure 5a) since the optical axis rotates, and therefore the light polarization becomes elliptical, which is described in detail [67,71,75]. This phenomenon limits the range of reliable registration of the phase retardation $\Delta \Phi \left(E\right)$ , change value in experiments. For example, it can be seen in Figure 5a that the magnitude of the phase retardation changes in electric field $\Delta \Phi \left(E\right)$ , which can be reliably recorded experimentally, does not exceed 10π in this case but generally the estimated value depends on parameters the DHFLC retarder.

Note, that the electrically controlled part $\Delta n_{eff}^{E}$ of the effective birefringence defined by Equation (11) is exactly proportional to the square of applied electric field, see Figure 5b. Consequently, this experiment also confirms the well-known orientational Kerr effect [33,34] in ferroelectric liquid crystals with a subwavelength helix pitch. But in addition to confirming the results of [33,34], let us add that the **Kerr** coefficient values calculated from the experimental data in Figure 5b according to Formula (11), significantly depend on the polarity of the applied field, namely, for the positive polarity of the field $K_{Kerr}=133\;nm/V^{2}$ , and for the negative one $K_{Kerr}=92\;nm/V^{2}$ . This difference is quite natural since we are dealing with the polar smectic C* phase but so far it has not been explained theoretically in any way. Nevertheless, the specified difference of **Kerr** constants must be considered when theoretically calculating the spectral modulation by DHFLC retarders.

For theoretical calculation of spectral modulation by any DHFLC cell, it is necessary to measure experimentally all the above parameters. For our research within the framework of this project, we chose the ferroelectric liquid crystal FLC-618, developed by Russian co-authors from Lebedev Physical Institute of RAS. The chemical structure of this FLC mixture was described earlier in [34] (but concentrations of chemical compounds have been changed a little for this research), and its measured parameters at 23 °C are as follows: $P_{s}=215\;nC/cm^{2}$ , $\;\theta_{0}$ **= 37 degrees**, $\chi_{G}=120$ at **0** < **E** **≤ 0.6 V/μm** and at the applied voltage frequency less than 60 Hz. The FLC layer thickness d of the prepared DHFLC cell is 107 μm.

**Figure 6:**
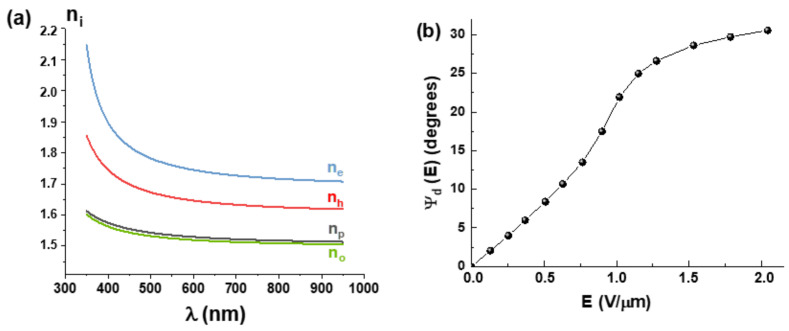
(**a**) Dispersions of refractive indices of the FLC-618; (**b**) the main optical axis rotation angle of the FLC-618 versus the applied electric field. Measurements were carried out at 23 °C.

Dispersions of all above mentioned refractive indices of the FLC-618 were measured using Mach-Zehnder interferometer according to a method grounded in [70]. The measurement results are shown in Figure 6a.

Based on the data presented in this Figure it is possible to calculate the dependence $\Delta n_{u}\left(\lambda \right)=n_{e}\left(\lambda \right)-n_{o}\left(\lambda \right)$ in the spectral range from 350 nm to 950 nm. In particular, $\Delta n_{u}$ = 0.24 at **λ** = 532 nm. At the same time, the range of reliable registration of changes in the electrically controlled phase delay **∆Φ (E)** of about 10π exhibits limitation (see Figure 5a) due to an increase of $\Psi_{d}\left(E\right)$ by more than 10 degrees (Figure 6b) when variations in $\Delta n_{eff}^{E}$ are within 0.024 (See Figure 5b), which is achieved at **E** ≈ 0.65 V/μm.

**Figure 7:**
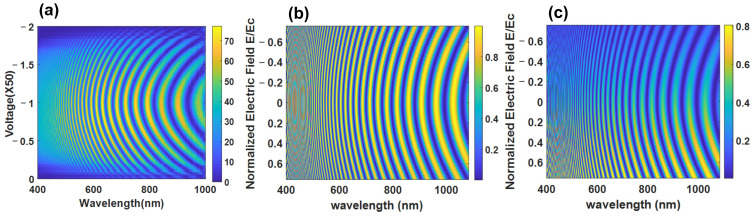
Spectral modulations of 131 µm DHFLC device while (**a**) represents the measured transmission for β=45 degrees; (**b**) shows the simulated transmission for β=45 degrees; (**c**) shows the simulated transmission for β=22.5 degrees while the color of the fringes represents the transmission coefficient value.

The choice of polarizer angle is crucial to get the best contrast in modulation as Figure 7 shows. By choosing polarizer angle β=45 degrees with respect to the LC optical axis at zero voltage, the contrast is better at low voltages and decreases for high voltages.

It is also seen from Figure 7c that asymmetry appears around the zero field when the angle β differs from the values 0, 45, and 90. The asymmetry appears obvious when  β is far from 45 degrees such as at 22.5 degrees. Since the cone angle $\theta_{0}$ stays constant with the applied field, the phase shift is less than the case of NLCs and the number of fringes stays high at higher voltages.

**Figure 8:**
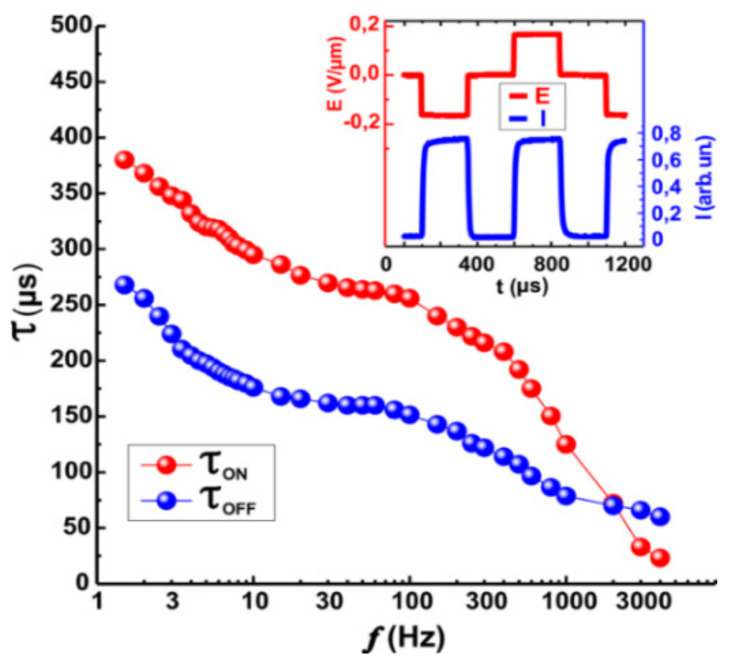
Frequency dependence of electrooptical response times $\tau_{ON}$ and $\tau_{OFF}$ of the DHFLC cell based on FLC-618, *d* = 107 μm, *T* = 23 °C. The inset illustrates the applied voltage and electro-optical response waveforms.

For small applied field **E << E_c_** and at a very low applied voltage frequency (within 10 Hz or even less), the DHFLC response time **τ** is defined by the relaxation time:

(12) $$\tau =\frac{\gamma_{\varphi }}{K_{\varphi }q_{0}^{2}}$$

where $\gamma_{\varphi }$ is the rotational viscosity defined in [20], $K_{\varphi }$ is the helix elastic modulus defined in [67], and ***q*_0_** *=* 2π/***p*_0_**. In this case, the effect of the field is insignificant due to the small helix distortion. It is also seen that the response time is independent of the device thickness *d*. For applied electrical field closer to the critical value, the helix is deformed strongly, and the response time is dependent on the applied field $\tau_{c}\propto \;E_{c}^{\epsilon }$ ( −1<ϵ<0 ) which started to behave like the surface stabilized FLC. If the field region near the $E_{c}$ , the pitch enlarges significantly $\left(p\gg p_{0}\right)$ but we do not work under this condition. In reality, the dynamics of the electro-optical response of DHFLC cells is determined by the dispersion **τ**(**f**) [77] (where **f** is the applied voltage frequency), which is only fragmentarily explained theoretically [74]. The results of measurements of this dispersion for FLC-618 are shown in Figure 8.

**Figure 9:**
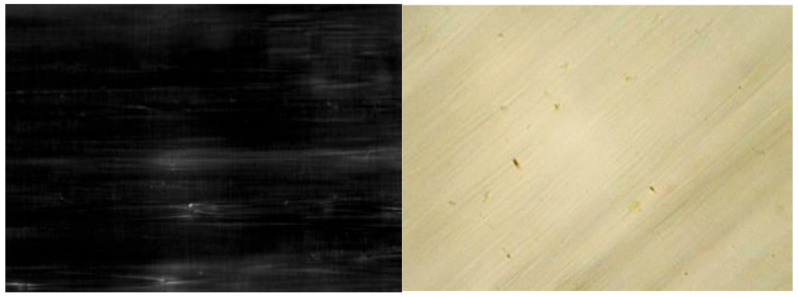
Micrographs of textures of a planar-aligned 107-µm thick layer of the FLC-618 helical nanostructure (*p*_0_ = 105 nm) in a polarizing microscope, image size 250 µm × 200 µm. Dark field on the left: the axis of the FLC helix is directed along the plane of the polarizer of the microscope, i.e., β=0 ; the bright field on the right when the axis is at the angle of β = 45°.

It is known [73] that the density of dislocations in planar-aligned layers of FLCs decreases with decreasing helix pitch, as well as with increasing of the FLC layer thickness, which makes it possible to produce electro-optical cells of a fairly high optical quality with the FLC layer thickness even more than 100 μm, see Figure 9.

**Figure 10:**
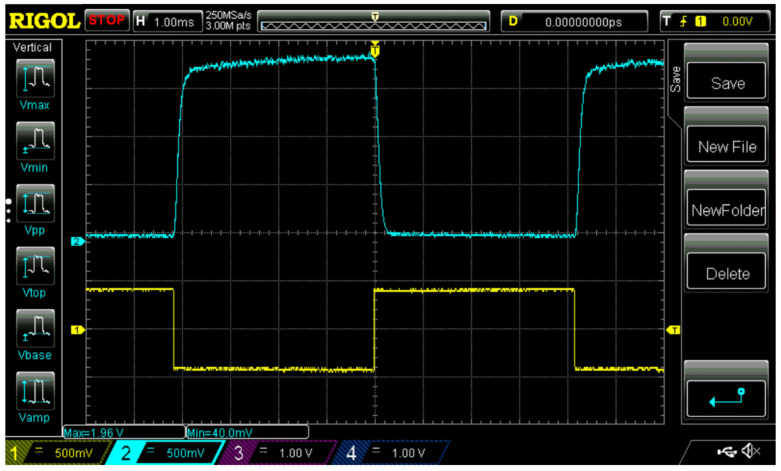
Oscillogram of the electro-optical response in white light (**upper curve**) of the planar-aligned FLC-618, the layer thickness of which in the electro-optical DHFLC cell is 107 μm. The lower yellow curve is the applied alternating voltage with the amplitude of ±40 Volts.

With the FLC-618 layer thickness of 107 µm, the contrast ratio of electro-optical modulation in white light is about 50:1 (defined as the ration between the maximum to the minimum levels), as can be seen from the oscillogram in Figure 10. Thus, the problem of the optical quality of thick DHFLC layers is not significant now.

## 3. Nematic versus DHF for Spectral Information Reconstruction

Based on the brief introduction of the two LC modes, the comparison between NLC and DHFLC retarders for spectral modulation and computational approach spectral imaging can be summarized in few points:

- Due to the spontaneous polarization and a very short helix pitch, DHFLC retarder has a shorter response time in the hundred or even tens of microseconds region while the response time of NLC is in the tens of milliseconds region. Moreover, increasing the retarder thickness in NLC causes an increase in the response time significantly, as Equation (4) shows, while the response time of DHFLC is thickness independent according to Equation (12).
- The best contrast in both LCs can be achieved when the retarder is oriented at 45 degrees with respect to the polarizer, as Figure 3 and Figure 7 show. Both LCs can provide good contrast for modulation with some privilege for NLC since the contrast is independent of the electric field’s value.
- Thick NLC retarders provide a large phase retardation shift, which leads to many fringes in the spectral modulation at zero voltage. By increasing the applied voltage, the total retardation of NLC decreases and the number of fringes decreases, as can be seen in Figure 3. This is considered an essential requirement to extract hyperspectral data with a smaller number of measurements [41]. In the case of DHFLC retarders, the phase retardation change is smaller by factor of 2–3. As a result, the number of fringes remains high at high voltages as Figure 7 shows. Hence, a larger number of measurements is required to compensate for that. However, the DHFLC fast response time could restrain this limitation.
- NLC retarders are operated at lower voltages with respect to DHFLCs because the former responds to the voltage while the latter responds to the field.
- Due to the various possible alignment materials and process simplicity, fabricating stable thick NLC retarders is more achievable than DHFLC.
- DHFLCs exhibit larger field of view than NLCs because the optic axis remains in the plane of the substrates.
- Both the NLC and the DHF have no hysteresis effects because the DHF is driven at voltages lower than the threshold.

## 4. Design and Simulation Results

### 4.1. The Inverse Scattering Approach

The inverse scattering or compressed sensing approach uses spectral multiplexing to extract the spectral information. Such spectral multiplexing can be made with optical devices that act as generalized spectral filters. According to this approach, rather than using spectral filters to filter out only a single narrow spectral band at each scanning step, more natural generalized spectral filters can be used to transmit or reflect a combination of non-uniformly weighted components from the entire spectral range. The transmitted or reflected light is given as a mathematical product between the input signal and the system spectral transfer function, which is integrated by a photosensor to produce a single spectrally multiplexed measurement. Repeated measurements using various filters provide enough information for reliable recovery of the spectral signal. The multiplexed spectral measurement process can be described by the integral:

(13) $$g\left(V\right)=\int_{0}^{\infty }\emptyset \left(\lambda ,V\right)\cdot f_{c}\left(\lambda \right)\cdot s\left(\lambda \right)\;d\lambda =\int_{0}^{\infty }\emptyset \left(\lambda ,V\right)\cdot f_{uc}\left(\lambda \right)\;d\lambda$$

This equation presents the mathematical expression for multiplex measurement. The $f_{c}\left(\lambda \right)$ is the spectral input signal (of the object or the seen). The function $\emptyset \left(\lambda ,V\right)$ is the spectral response of the modulator, in our case, the spectral transmission of the liquid crystal retarder. The spectral encoding function $\;\emptyset \left(\lambda ,V\right)$ is dependent on the voltage applied on the cell. For each selection of **V**, the encoding is changed and a $g\left(V\right)$ measurement is recorded. Here $s\left(\lambda \right)$ defines the spectral response of the photosensor or the photo diode. Generally, the spectral information is given by the right side of Equation (13). The actual input signal is given as $f_{uc}\left(\lambda \right)=f_{c}\left(\lambda \right)\cdot s\left(\lambda \right)$ . The presentations in Figure 3 and Figure 7 are the spectral response functions $\emptyset \left(\lambda ,V\right)$ for each corresponding device or geometry.

To present the system in the form of Equation (13) some discretization of the sensing operation model is needed. By using a discretization of the spectrum, we can write Equation (13) in the following form:

(14) $$g\left(V\right)\approx \sum_{\lambda =1}^{N_{\lambda }}\emptyset_{\lambda }\left(V\right)\cdot f_{\lambda }\cdot s_{\lambda }=\sum_{\lambda =1}^{N_{\lambda }}\emptyset_{\lambda }\left(V\right)\cdot f_{\lambda ,uc}$$

As the voltage that is applied on the cell is with finite voltage steps, a discretization procedure is associated with a finite number of filter configurations. If we let **V** take discreet values, we can rewrite Equation (14) as:

(15) gk=∑λ=1Nλ∅λk·fλ,uc → gMλ=ΦMλNλ·fucNλ

This form is the standard form of matrix vector multiplexing. It can also be written in terms of the calibrated spectral signal and the spectral responses of the optical system and the optical sensor as:

(16) $$g=\Phi f_{uc}=\Phi Sf_{c}\;f_{c}=S^{-1}{\hat{f}}_{uc}$$

$$f_{c}=S^{-1}{\hat{f}}_{uc}$$

where the **S** matrix represents the system spectral responses, or in other words **S** is the transformation from the input signal to the un-calibrated signal measured in the system. **S** is a square diagonal matrix in which the entries outside the main diagonal are all zero and the entries on the main diagonal have positive values. It is guaranteed that **S** has an inverse $S^{-1}$ , which is also a diagonal matrix obtained by replacing each element in the diagonal with its reciprocal.

### 4.2. System Simulation of Multi-Band Pass Filter

**Figure 11:**
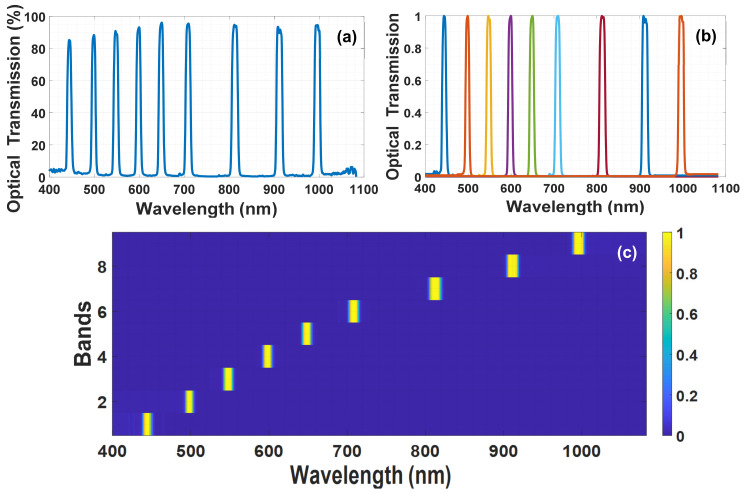
(**a**) This graph shows the spectral transmission of a multi-channel spectral passive filter. The filter can be designed to transmit the wavelengths according to the specific application. (**b**) This figure depicts the breaking of the spectral transfer function of the filter to a set of spectra that can form any spectral signal which will pass through the filter. It should be noted that the spectral filter contains 9 spectral windows and now this is displayed as 9 different spectra (in different colors) that together form any spectral signal that reaches the detector. (**c**) Transpose matrix transition from channel weights to spectrum, in other words this is the matrix form of (**b**).

In [41,78,79] August et al. showed a way to capture and reconstruct high number of spectral bands for spectroscopy or spectroscopic imaging. However, in our case and in many other applications, the necessary spectral information does not have to be presented by large number of spectral channels, meaning multispectral information is adequate rather than hyperspectral. The multispectral case means sampling the signal over a smaller number of exposures, which will allow a faster measurement of the spectral information. Our proposition [80] is to use a passive optical filter giving several pass bands combined with the LC spectral modulator so that a priori our sensing matrix will have limited size and many zeros. This way the number of voltages required will be minimized and the overall spectrum extraction will be simplified and become faster. The passive filter may then be chosen to give the relevant wavelengths for the specific application. In an ideal case when the passbands are delta functions, it is easy to see from the analysis above that the number of voltages required equals to the number of wavelengths that passes through the passive filter. Because in reality the pass bands have some spectral width, larger number of voltages are required. Generally, the spectral signal is described using large number of samples. But in our case, we describe the spectral signal with a limited number of channels. Hence the spectral signal is described by means of a small number of weights, which are multiplied by the spectral windows of the passive filter. In this description, each spectral channel transmitted through the filter can transmit a certain light intensity, and the ultimate goal is of course to estimate what the light intensity was through each spectral window. Figure 11a shows the optical transmission of our multiband pass passive filter (customized spectral filter by IRIDAN Spectral Technologies Ltd., Ottawa, Canada).

According to the mathematical description given in the previous section, now the spectral signal is represented by a product of weights in a transition matrix:

(17) $$f_{c}=M\alpha$$

$$g=\Phi f_{uc}=\Phi SM\alpha$$

The matrix **M** represents the transition from channel weight to spectrum. The Alpha vector α is the vector of weights in each spectral channel, in other words it is a vector of weights in each channel where each channel presents a wide or narrow wavelength range according to the design of the spectral filter used. To assemble the matrix **M**, one has to break down the spectral transfer function of the multi-channel filter shown in Figure 11a into several separate spectral transitions, each containing one band pass, and present each measured signal as a product of weight in the corresponding spectral band as shown in Figure 11b. To present the process in a matrix form, we can turn each band shown in the previous figure into a column in matrix **M** and get the **M** matrix as shown in Figure 11c.

The sensing process is given according to the following equations:

(18) $$g=\Phi f_{uc}+\varepsilon =\Phi SM\alpha +\varepsilon =\gamma \alpha +\varepsilon$$

γ=ΦSM

Here ε is some noise and we are looking for the sparsest solution for α . In this work we used Orthogonal Matching Pursuit Algorithm (OMP) [81,82] to find α and $f_{c}=M\alpha$ . Orthogonal Matching Pursuit Algorithm is a greedy compressed sensing recovery algorithm, which selects the best fitting column of the general sensing matrix γ=ΦSM in each iteration. A least squares optimization performed in the subspace spanned by all previously picked column vectors. This method is in general less accurate than other algorithms, however it has a lower computational complexity. In general, during the recovery process, we evaluate the vector α and recover $f_{uc}=M\alpha$ . by knowing and using matrix S we can estimate $f_{c}$ by using $f_{c}=S^{-1}{\hat{f}}_{uc\;}$ .

### 4.3. Simulations Outputs

In this section, a number of results from simulations made to obtain an estimate of various parameters in the sensing system are presented. The simulations show results for the following parameters, signal-to-noise ratio, number of exposures (number of operating voltages of the LC cell), and the thickness of the liquid crystal cell. We have chosen to present the capability and performance of the system in terms of PSNR. Peak signal-to-noise ratio (PSNR) is an accepted quantitative measure of the quality of recovery or system performance. The PSNR is defined as:

(19) $$MSE=\frac{1}{mn}\overset{m-1}{\underset{i=0}{\sum }}\overset{n-1}{\underset{j=0}{\sum }}{\left[I\left(i,j\right)-K\left(i,j\right)\right]}^{2}$$

$$PSNR=20log_{10}\left(\frac{MAX_{I}^{2}}{MSE}\right)$$

Here **I** is the spectral signal and its noisy approximation **K** while **MAX_I_** is the maximum possible value of the spectral signal. High values of **PSNR** represent high refinement in spectral signal reproduction and low values represent low ability.

#### 4.3.1. Nematic Case

**Figure 12:**
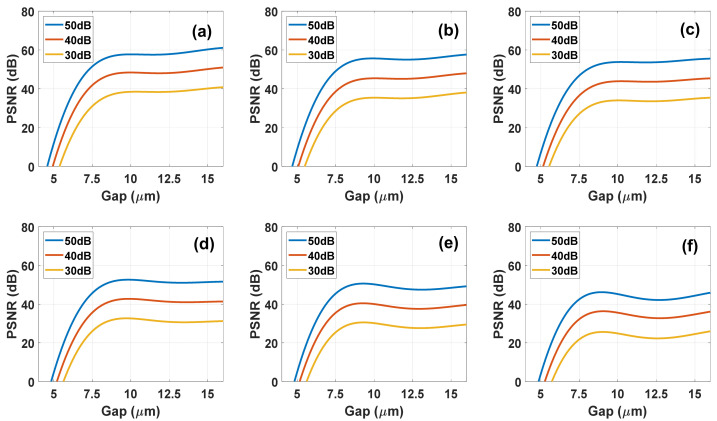
Simulation results of the reconstruction quality measured in PSNR, as function of the NLC E7 gap at different SNR as indicated for each curve values and different number of Voltages: (**a**) 56; (**b**) 28; (**c**) 19; (**d**) 14; (**e**) 12; (**f**) 10.

The results for NLC system are shown in the Figure 12, each sub-illustration describes the system performance for a different number of exposures or in other governances the number of voltages used for whispering. In addition, each curve describes a different signal-to-noise ratios of 50 dB, 40 dB, and 30 dB.

**Figure 13:**
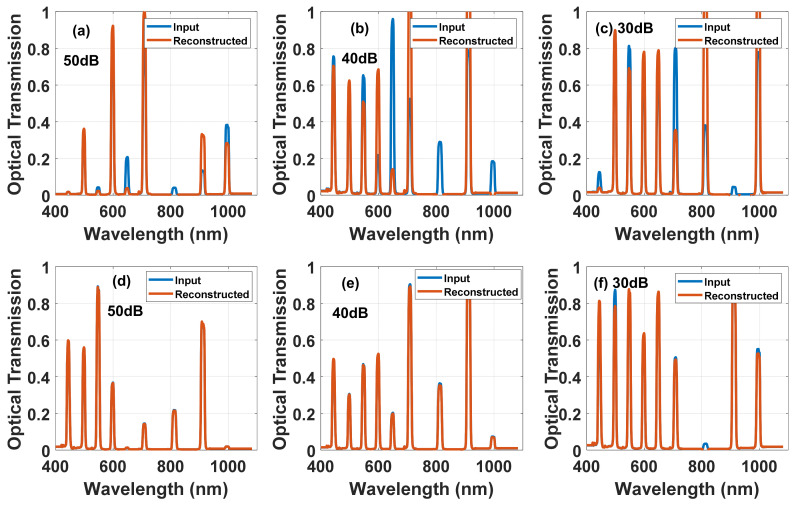
Simulated reconstructed spectra with the input signal is the blue curve and the red is the reconstructed ones using two different NLC E7 gaps, 6 µm for (**a**–**c**) while 12 µm for (**d**–**f**) and different SNR values in dB: (**a**,**d**) 50; (**b**,**e**) 40; (**c**,**f**) 30. The number of voltages is 19.

From the results of these simulations, it is seen that using a nematic cell with a thickness of about 10 microns the quality of the reconstruction is almost unchanged. In addition, it can be noticed that the number of exposures (number of voltages) affects the quality of the reconstruction. The results show average values from the raw simulation results, it can also be said that as the value of the recovery quality decreases the probability of error also increases. To illustrate this, in Figure 13 we present the input spectrum and reconstructed one at two different NLC cell thicknesses and different noise levels. It clearly shows that 10-µm thick device with 50 dB SNR is adequate to fully reconstruct the full spectrum of 9 bands.

#### 4.3.2. DHFLC Case

**Figure 14:**
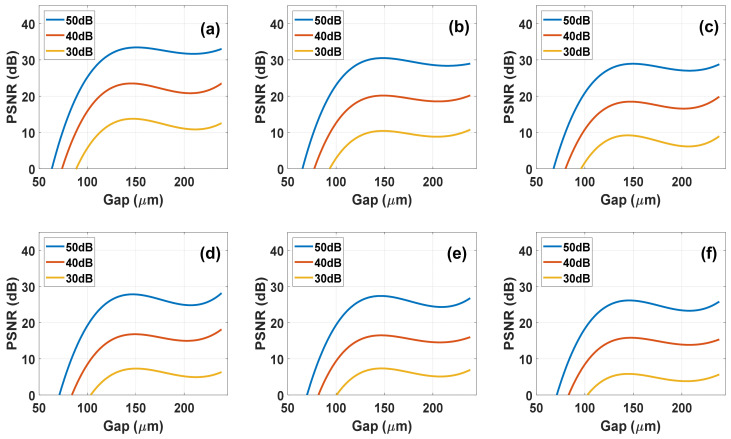
Simulation results of the reconstruction quality measured in PSNR, as function of the DHFLC cell gap at different SNR as indicated for each curve values and different number of voltages: (**a**) 151; (**b**) 76; (**c**) 51; (**d**) 38; (**e**) 31; (**f**) 26.

**Figure 15:**
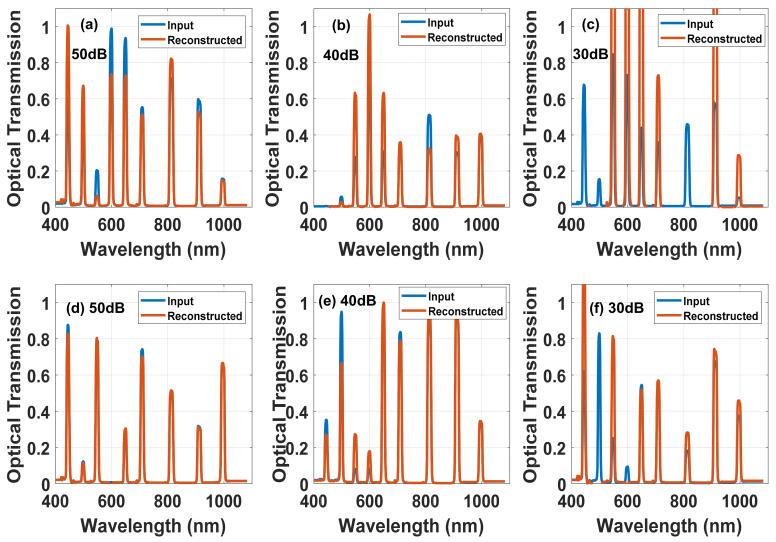
Simulated reconstructed spectra with the input signal is the blue curve and the red is the reconstructed ones using two different DHFLC-618 gaps, 110 µm for (**a**–**c**) while 153 µm for (**d**–**f**) and different SNR values in dB: (**a**,**d**) 50; (**b**,**e**) 40; (**c**,**f**) 30. The number of voltages is 51.

A DHFLC-based system has a different sensing matrix. For the reconstruction to be possible, a larger cell thickness and a greater number of exposures are needed than those required in the NLC system because the spectral modulation and contrast are less. The Figure 14 below shows the PSNR versus DHFLC cell gap at different voltage numbers used. Compared to the NLC case, it is clear that the reconstruction in the NLC is of higher quality, however considering the fact that the DHFLC is faster by nearly 2–3 orders of magnitude, for certain applications the use of DHFLC is feasible. Figure 15 shows that 131 µm thick device with 50 dB SNR is adequate to fully reconstruct the full spectrum of 9 bands.

### 4.4. Viewing Angle Effect on Inverse Scattering Results

**Figure 16:**
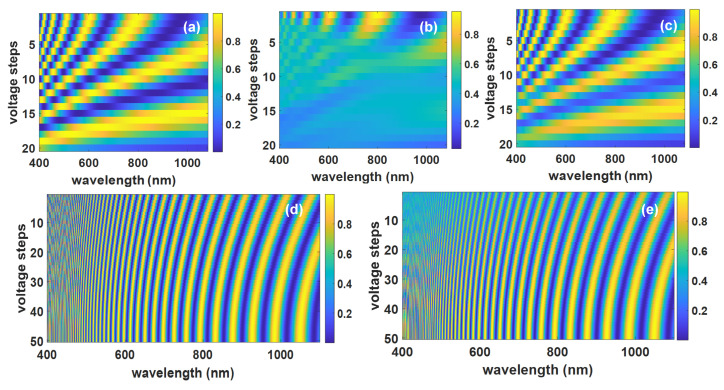
Simulated transmission matrix for different incidence angles: (**a**) NLC E7 at 0 deg; (**b**) is the averaged modulation of anti-parallel NLC E7 at 20° light-cone; (**c**) is the averaged modulation parallel NLC at 20° light-cone; (**d**) is the DHF FLC-618 at 0 deg; (**e**) is the averaged modulation of DHFLC at 20° light-cone.

As mentioned previously, DHFLCs exhibit a larger field of view than NLCs in terms of spectral modulation. To evaluate the effect of the viewing angle on inverse scattering results, we calculated the spectral modulation from 131 μm  DHFLC retarder,  10 μm NLC anti-parallel and parallel retarders. The calculations were prepared for the range of −20° to 20° incident angles. Figure 16a,d shows the simulated transmission matrix at 0 normal incidence on the NLC and DHFLC retarders, respectively. Figure 16b,c,e shows the spectral modulation average for 20° light cone of anti-parallel NLC, parallel NLC, and DHFLC retarder. It is seen from Figure 16 that the contrast is reduced significantly for anti-parallel NLC while it is affected less for parallel NLC and DHFLC retarders.

**Figure 17:**
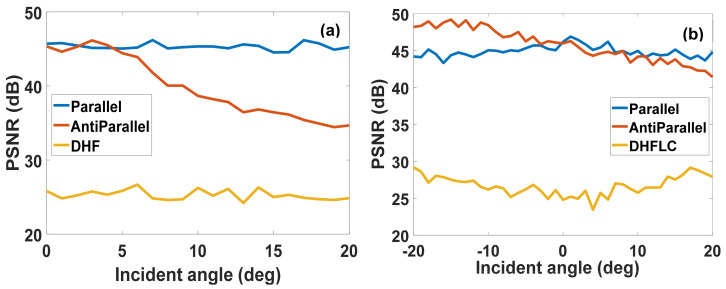
Reconstruction quality measured in terms of PSNR versus the incidence angle: (**a**) is for cone of light; (**b**) is for off-axis collimated beam.

The effect of the viewing angle is evaluated by calculating the PSNR of the reconstruction based on transmission matrices at −20° to 20° incident angles on two cases: (1) an average of modulation of a cone of light beam over the angles and (2) a collimated light beam, while Figure 17a,b shows the results, respectively. It is seen in both cases that PSNR calculated based on matrices of DHFLC and parallel NLC maintain the same level over wide light incidence angle range while it decreases significantly for anti-parallel NLC in the case of a cone of light case due to the asymmetry that also appear in Figure 17b.

## 5. Conclusions

The parallel spectroscopic extraction using spectral modulation and computational algorithms was examined for the purpose of obtaining faster extraction speed. Two approaches are investigated: (i) Using nano helical ferroelectric LCs in the DHF mode as it gives response times in the range of 100 µs without dependence on the thickness contrary to NLCs; and (ii) using a passive multiple passband filter which gives wavelengths designed for the specific application, thus minimizing the sensing matrix dimension, and simplifying the reconstruction algorithm. A passive filter giving 9 pass bands covering the visible and near infrared ranges was used. It was shown that NLC devices with 10 µm thickness and 19 voltages are adequate to reconstruct the 9 bands with PSNR around 60 dB. Considering fast NLCs with 10 ms response time, the reconstruction time will take around 100 ms to 150 ms. To monitor PPG signals, we need to grab at least ten spectra during the heartbeat, meaning the requirement on the reconstruction time needs to be maximum 100 ms, hence the fastest nematic LC device is marginal for this application. Nevertheless, for other applications the NLC might be enough and here we proposed a methodology to allow it. On the other hand, the DHFLC needs to be thicker (around 120 µm minimum) showing PSNR of 40 dB, however it is faster by a factor of 20–50 and therefore for sure it is enough for PPG multispectral signal extraction. This work exposes a methodology with high potential for fast multispectral imaging which can have many applications in medicine, food inspection, and industrial processes inspection. Experimental verification of these findings is ongoing and will be published shortly.

## Figures and Tables

**Table 1:** Selection of applications from different fields that use only small number of wavelengths.

Application	Spectral Bands Used (nm)	Tuning Methodology	FWHM (nm)	Ref
Skin cancer diagnosis	440, 460, 490, 510, 530, 550, 710, 780, 790	LCTF	-	[43]
Skin chromophores mapping	700, 750, 750, 800, 850, 1000	Filter wheel	40	[44,45]
Skin cancer detection	500–700 in 10 nm steps	LCTF	8	[46]
Melanoma and Nevi evaluation	540, 650, 950	Passive filters	15	[47]
Skin cancer detection with smartphone	450, 470, 500, 530, 580, 660, 810, 940	LEDs	20	[48]
Skin hemodynamics	RGB	Camera filters	>60	[49]
Oral cavity detection	627, 512, 447, 573	LEDs	20	[50]
Moldy apple core detection	425, 455, 515, 615, 660, 700, 850	Pixelated spectral sensor	-	[51]
Minced beef evaluation	515, 595, 650, 880	Spectrograph	-	[52]
Assessment of fruits and vegetables	Different combinations of wavelengths in the range 435–950	Commercial instruments	-	[53]
Blood glucose measurement	9259, 10526	CO_2_ laser	<1	[54]
Two or three wavelengths pulse oximetry	805 and other wavelength, 767&811, three wavelengths in the range 760–900	LEDs	18	[55]
Retinal oximetry	570, 600 or the blue-green	Lasers, LEDs	-	[56,57]
Remote sensing, spectral LiDAR	8 wavelengths covering 470 nm–830 nm range	Filters	10–50	[58]

## Data Availability

Data is available upon request.
